# Shape dependent physical mutilation and lethal effects of silver nanoparticles on bacteria

**DOI:** 10.1038/s41598-017-18590-6

**Published:** 2018-01-09

**Authors:** Debashish Acharya, K. Malabika Singha, Piyush Pandey, Bidhan Mohanta, Jina Rajkumari, L. Paikhomba Singha

**Affiliations:** 1 Department of Physics, Assam University, Silchar, 788011 India; 2 Department of Microbiology, Assam University, Silchar, 788011 India

## Abstract

In this report, spherical silver nanoparticle (AgNP-sp) and rod-shaped silver nanoparticle (AgNR) were prepared by chemical reduction method and their antibacterial activity against various Gram-positive and Gram-negative bacteria had been evaluated for their efficiency. Minimal inhibitory concentration (MIC) tests were conducted to study the antibacterial properties, and substantiated with killing kinetics of silver nanoparticles (AgNPs). The study revealed that both AgNP-sp and AgNRs are good antibacterial candidates. Bacterial sensitivity to nanoparticles (NPs) was found to vary depending on microbial species. Disc diffusion studies revealed the greater effectiveness of AgNP-sp and AgNR against *Klebsiella pneumoniae* AWD5 at the doses of 249 and 392 µg. The dose dependent activities of prepared NPs were also observed on the batch studies of disc diffusion and MIC with various strains. The optical and morphological structures of NPs were analyzed by UV-visible, XRD, FE-SEM and TEM. Further, FESEM of bacterial culture treated with AgNPs confirmed antibacterial activity of NPs by showing rupture of bacterial cell wall. Also, the genome of test organism was found to have *CusCFBA* and *CusRS* operons. The killing kinetics confirmed that the death rate of *K. pneumoniae* was higher against AgNP-sp as compared to AgNR.

## Introduction

The metal nanoparticles such as silver, gold are becoming indispensable in various fields because of their vast applications against various diseases and environmental applications. Since ancient times, silver had been used as antiseptic and antimicrobial agents against various Gram-positive and Gram-negative bacteria^1–3^. There are reports that confirm silver ions and silver based products are highly toxic to various microorganisms, which include about 16 species of bacteria. Further, the shapes of nanoparticles (NPs) have invited much attention because of variation in their effects on bacteria. It may be speculated that the toxicity depends upon the size, shape and morphology. For smaller size of NPs larger is the surface-volume ratio and more is their toxicity, and so the probable mode of interaction with bacterial surface is also greater^4–8^. Earlier, spherical and triangular silver nanoprisms had been compared and triangular silver nanoprisms were reported to have higher antibacterial activity against *Escherichia coli* and, attributed to the sharp edges and vertexes of the nanoprisms^9^. Similarly, different aspect ratio of AgNRs had been found to have antibacterial effects against *Bacillus subtilis* and *E. coli* ^10^. Though some of the conclusions were based on measuring zone of inhibition (ZOI) diameters^9^. Earlier reports emphasize dependence on morphology and distribution with reactive faces of the AgNPs for antibacterial activity. In another report, enhanced antibacterial action of Ag nanosphere was compared to triangular nanoplates, and enhanced antibacterial activities of Ag nanosphere were attributed to the greater contact with bacteria compared to nanoplates^11^.

Hong *et al*. compared the antibacterial activities of Ag nanocubes, spheres and wires against *E*. *coli* ^12^, and found that spheres are more effective in the inactivation of *E. coli*. Further, based on TEM analysis, it was concluded that the low specific surface area of Ag nanowires was the probable reason for weakening of antibacterial activity. Due to the large effective specific surface area, the Ag nanocubes and spheres achieved closer contact with bacterial cells and cause more damage to the bacterial cell membrane^12^.

Though some bacteria are known to be resistant for silver ions due to presence to resistant determinant in genome that facilitate efflux-mediated resistance for metal ions^13^. As it is known, that each bacterium has specific regulatory mechanisms that control different activities of membrane transporters. Proteins from several transporters families had been recognized to have significant role in efflux of metal ions. β-barrel proteins from general bacterial porin (GBP) family are known to catalyzes the energy-independent movement of polar solutes, including some non-essential metal ions, across the outer membrane of Gram-negative bacteria. Heavy metal resistance determinant on chromosomes and mobiles genetic elements encodes arranges of membrane transporters that transports specific toxic metals out of the cell. The expression of these systems is controlled by ultra-sensitive regulators^14^. Considering all these, in this work, both size and shape dependent antibacterial property of AgNPs has been investigated, by synthesizing two different shaped AgNPs – spheres and rods, against Gram-positive and Gram-negative bacteria at different concentrations.

## Result and Discussion

**Figure 1:**
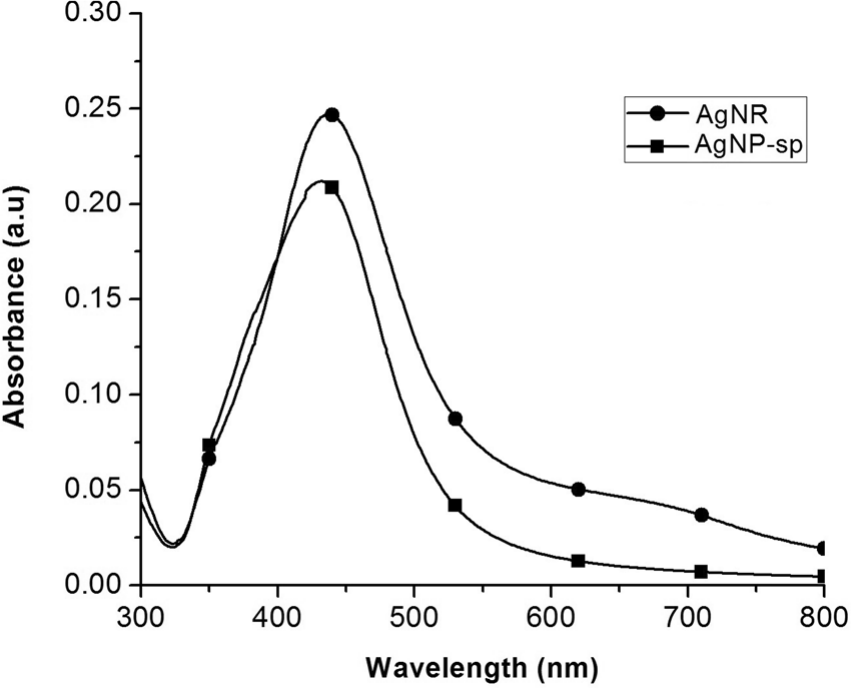
UV-Vis absorption spectra of AgNP-sp and AgNR in aqueous solution.

The UV-Visible absorption spectroscopy is one of the most widely used analytical techniques for the structural characterization of the synthesized NPs. The absorption spectra of AgNP-sp and AgNRs reduced by citrate is shown in Fig. 1. The surface plasmon resonance (SPR) peak for AgNP-sp was found at 430 nm. Figure 1 (AgNR) exhibits the two plasmon mode at different intensities such as longitudinal at peak position 437 nm and transverse mode at 346 nm which clearly reflects the anisotropic shaped NPs. The more intense longitudinal band arises due the coherent dipole oscillation along the long axis of nanorod, while the less intense transverse band is due to dipole oscillation perpendicular to the short axis of the nanorod^10^. In the present synthesis technique, role of citrate is supposed to be important because it reduce and stabilize the AgNP-sp and AgNR for a long time^8,15^.

As colloidal AgNPs possessed negative charges by the absorption of citrate ions, an electrostatic repulsive force worked among the particles, which prevented the particle aggregation for a long time^16^. In case of nanorod synthesis, the addition of NaOH causes the further oxidation of small particles into Ag^+^ ions and results in transformation of anisotropic shape from spherical shape. The presence of NaOH is also important for production of high yield nanorod. It has been reported that different amount of NaOH had a direct influence in the reaction for the production of silver nanowire^17^. The mechanism of nanorod formation in the work can be best explained by Ostwald ripening^17,18^ and the different capping action of citrate. The ripening involves the formation of larger NPs by the aggregation of smaller NPs when the solution is heated at higher temperature and this will be further capped with citrate present in the solution. At room temperature, the layers of citrate produce uniformly stabilized particles, but at elevated temperature, the equilibrium constant for citrate binding to certain crystal faces of silver begin to differ that leads to selective loss of citrate on specific crystal faces and thus allowing the NPs to grow along only one axis^17,18^. Reportedly, the ripening processes for colloidal silver dispersion had been suggested to take place at a temperature 85 °C^18^.

**Figure 2:**
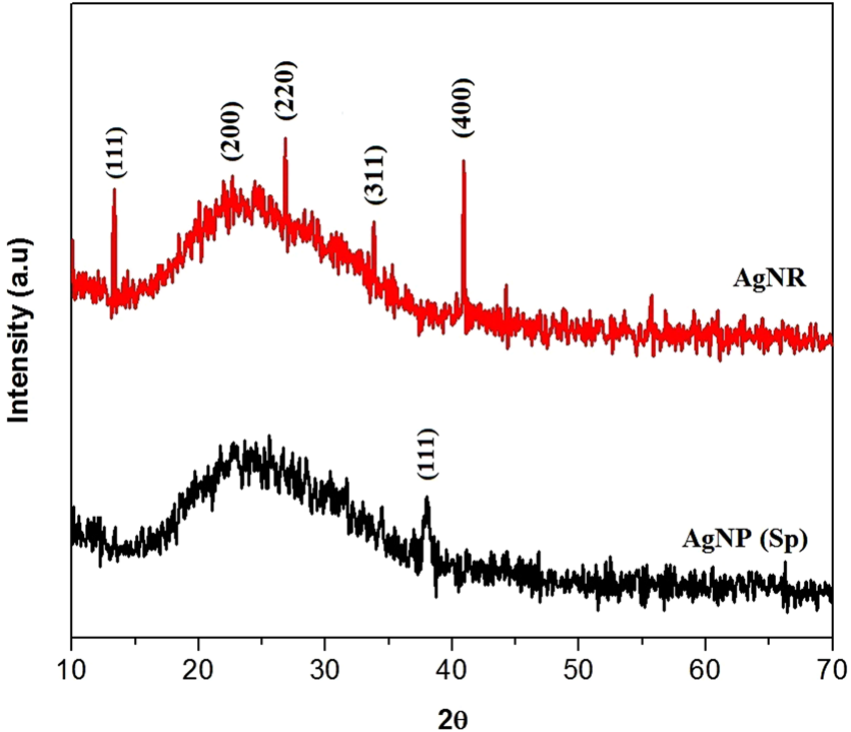
XRD spectra of AgNR and AgNP-sp.

**Figure 3:**
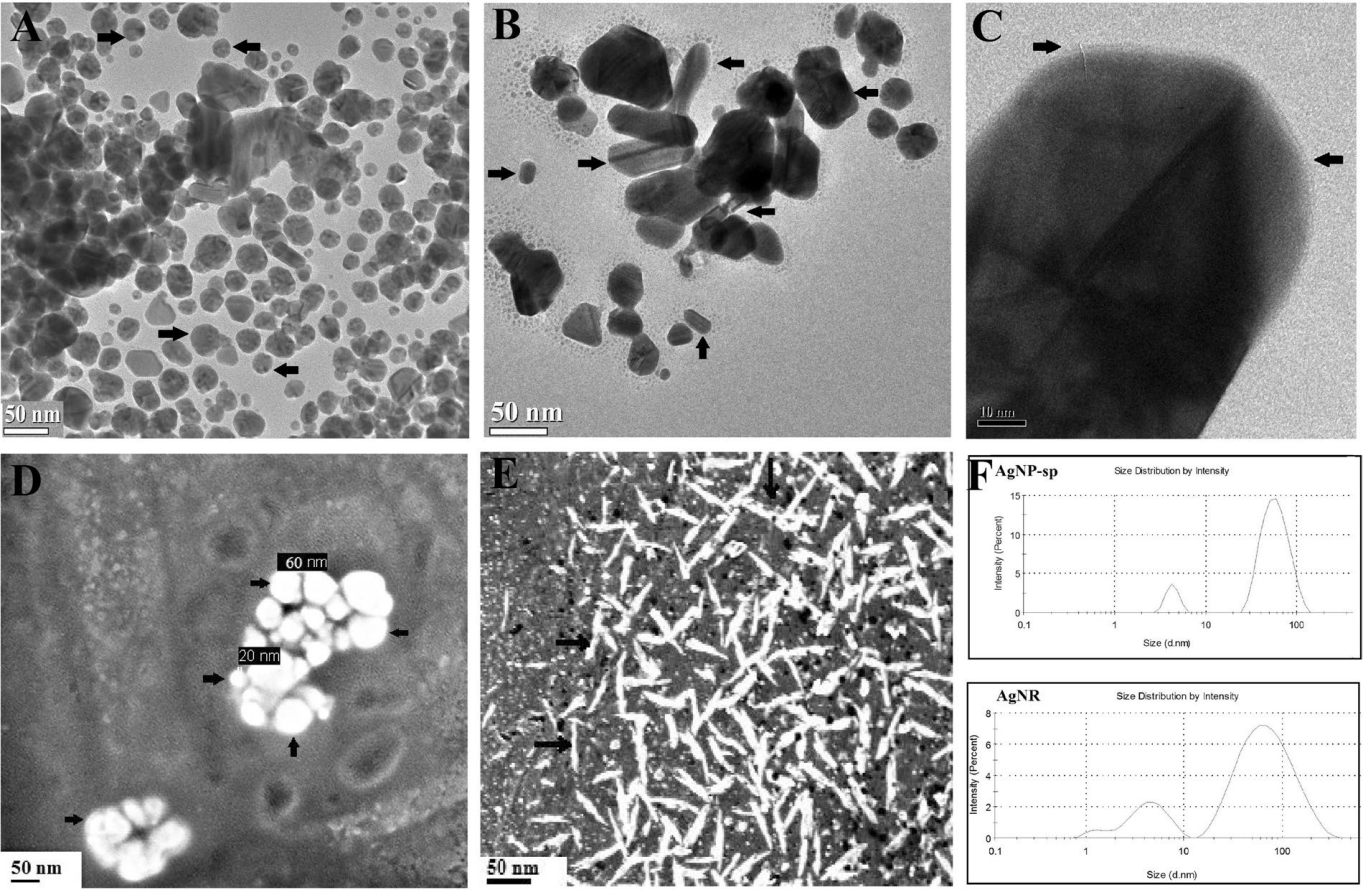
TEM images of spherical AgNPs (**A**), rod AgNPs (**B**), and HR-TEM images of nanorod showing sharp edges (**C**). FE-SEM images of spherical (**D**) and rod shaped AgNPs (**E**), The size distribution profile of AgNP-sp and AgNR by the DLS techniques (**F**).

Crystallographic data obtained from XRD analysis revealed that both AgNP-sp and AgNRs were of face cubic centre (fcc) structure (Fig. 2). The peak appeared for AgNP-sp at 2θ = 38.02° corresponds to crystal plane (111) (JCPDS File No: 03-0921) and those for AgNR are 17.21°, 22.62°, 26.82°, 33.78° and 40.89° corresponding to the plane (111), (200), (220), (311) and (400) respectively (JCPDS File No: 01-1164). It was found that the AgNPs having the plane contain high atomic density of electrons and are known to be highly reactive^4^. In this study, the antibacterial activities against different species were found to be present in both AgNP-sp and AgNRs, better being in AgNP-sp. This may be due to the interaction of the bacterial surface with the (111) planes of AgNPs. The prepared samples were further analyzed by FE-SEM and TEM to reveal the morphology of AgNPs and dynamic light scattering (DLS) technique to obtain the size distribution of NPs (Fig. 3). The morphology indicates that some spherical NPs agglomerated by small grains of mean size 40–50 nm. However, some particles of mean size more than 60 nm were also seen, which may be because of aggregation of the smaller particles formed during synthesis process^18^ (Ostwald ripening) (Fig. 3A,D). For AgNR, the relative length was measured, the shortest of which was found as 20 nm and the longest more than 90 nm (Fig. 3B,E). The width of the rod was found, on average 20 ± 6 nm. However, some spherical along with triangular NPs were also seen in TEM images, though their density was negligible as evident from FE-SEM image (Fig. 3E). Occurrence of few spheres and triangles may be due to the different capping action of citrate at higher temperature^18^.

**Figure 4:**
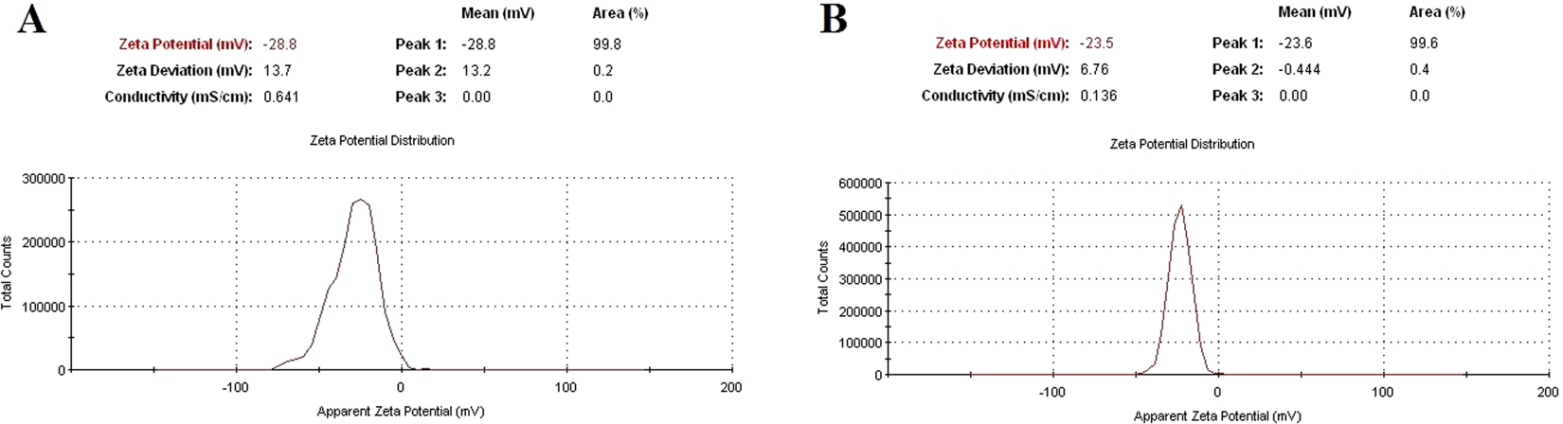
Surface zeta potential analysis of (**A**) AgNP-sp and (**B**) AgNR.

The normal size distribution of the two (rods and spheres) AgNPs obtained by DLS analysis were found in correlation with FE-SEM and TEM studies. The sharp edges of NPs were visible in rods (Fig. 3B) and spheres (Fig. 3A). Further, the magnitude of electrostatics charge potential at the electrical double layer surrounding AgNPs was measured by zeta potential. According to zeta potential analysis theory, the magnitude of zeta potential data indicates the stability of the NPs in an aqueous environment^19,20^. Here, the values of zeta potential of AgNP-sp and AgNR were found as −28.8 mV and −23.5 mV respectively (Fig. 4). The negative value with high magnitude indicated the highly stable environment of the AgNPs. This analysis also suggests the presence of strong electric charge on the particle surface hindering the aggregation of NPs^20^.

MIC values of AgNP-sp and AgNR was estimated against different bacteria as given in Table 1. The antibacterial activity of AgNP-sp was recorded significantly higher (P < 0.05) than AgNR. Moreover, the results also suggested that the antibacterial activity was more prominent against tested Gram-negative bacteria, as compared to Gram-positive bacteria.

**Table 1:** MIC values of AgNP-sp and AgNR against different bacterial strains.

Bacterial Strain	Strain no.	AgNP-sp concentration in MIC value (µg/ml)	AgNR concentration in MIC value (µg/ml)
**Gram-positive**			
*S. aureus*	ATCC 25923	190	358
*B. subtilis*	AST5-2	195	350
**Gram-negative**			
*P. aeruginosa*	AL2-14B	188	348
*K. pneumoniae*	AWD5	184	320
*E. coli*	ATCC 25922	190	340

In MIC test, the bacteria in control remained in stationary phase after 3–4 hours, whereas the growth of *K. pneumoniae* AWD5 was inhibited at 184 µg/ml, and 320 µg/ml concentration of AgNP-sp, and AgNR respectively. Similarly, the MIC values of AgNP-sp and AgNR for *E. coli* ATCC 25922 was 190 µg/ml and 340 µg/ml, for *Pseudomonas aeruginosa* AL2-14B 188 µg/ml and 348 µg/ml and for *S. aureus* ATCC 25923 was 190 µg/ml and 358 µg/ml, for *B. subtilis* AST5-2 was 195 µg/ml and 350 µg/ml respectively. These values are significantly lower than 9000 µg/ml MIC value for a commercially manufactured AgNPs, where the antibacterial test had been carried out at initial cell concentration of 10^5^ CFU/ml^21^. In another study, Agnihotri *et al*. synthesized citrate stabilized AgNPs of different sizes ranging from 5–100 nm and studied their antibacterial effect against Gram-positive (*S. aureus* NCIM 5201) and Gram-negative bacterium (*E. coli* MTCC 443), and found enhanced antibacterial effectiveness of smaller sized nanoparticles (5–10 nm). The MIC values for different sized AgNPs (5–100 nm) were found from 20 to 200 µg/ml at initial bacterial concentration of 10^5^ to 10^6^ CFU/ml^22^. In a different study, MIC of citrate stabilized AgNPs was found to be 125 µg/ml for *E. coli* ATCC 8739, where initial bacterial concentration was 10^5^ to 10^6^ CFU/ml for 20–65 nm sized AgNPs, which is similar to results of present work^23^.

**Figure 5:**
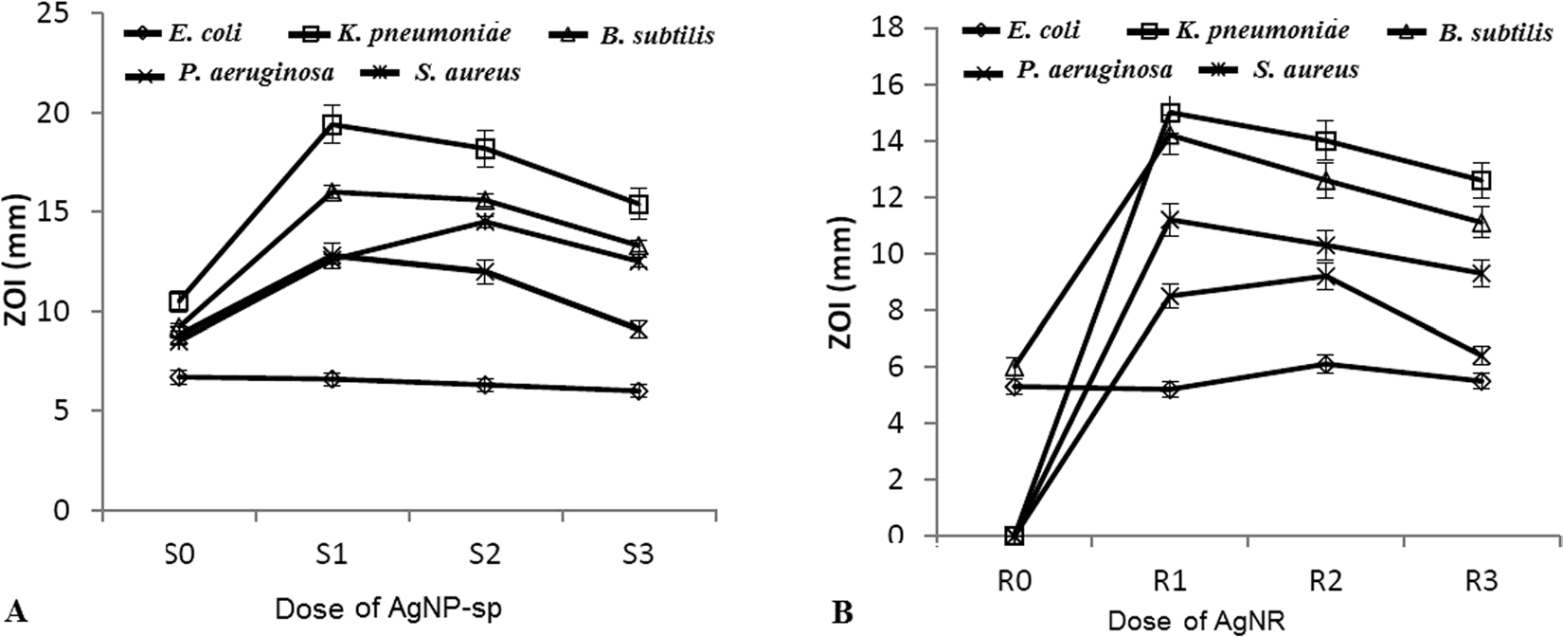
Antibacterial activity of AgNPs against different bacteria (*E*. *coli* ATCC 25922, *S. aureus* ATCC 25923, *B. subtilis*, *P. aeruginosa*, *K. pneumoniae* AWD5) at different concentrations of (**A**) AgNP-sp and (**B**) AgNR. 255, 249, 242 and 230 µg assigned as S0, S1, S2 and S3 for AgNP-sp and 399, 392, 380, 364 µg for AgNR were assigned as R0, R1, R2 and R3 respectively.

**Figure 6:**
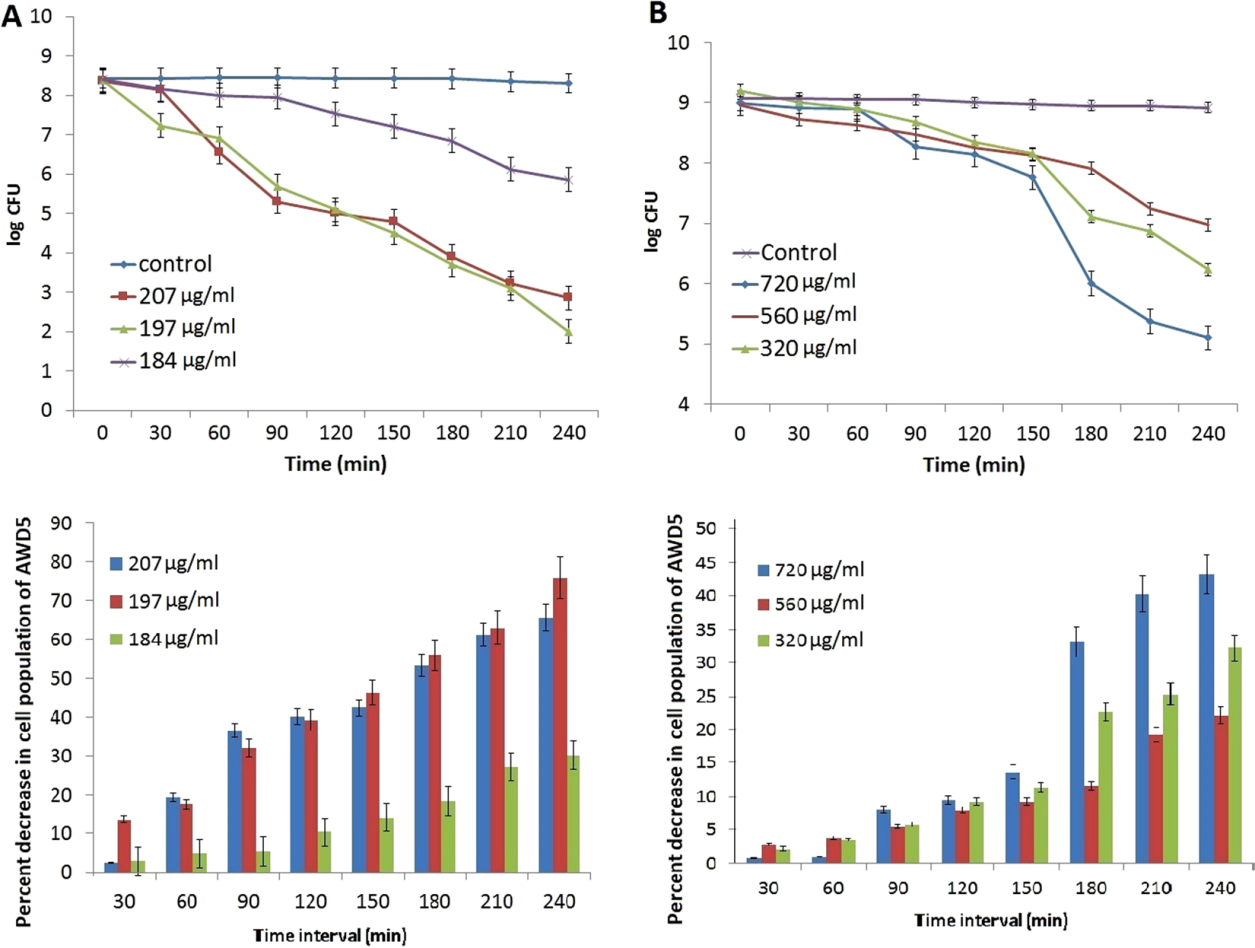
Killing kinetics *of K. pneumoniae* AWD5 exposed to (**A**) AgNP-sp at concentrations of 184–207 µg/ml and (**B**) AgNR at a concentration of 320–720 µg/ml. The percent decrease in cell population at different time intervals (0–4 h) is also given below the respective figure. Error bar represents the standard error of mean for three independent assessments.

Herein, the AgNPs of both spherical and rod shaped showed excellent antibacterial effect at different concentrations. We observed ZOI at different doses of Ag nanorod and spheres by disc diffusion test (except R0, 399 μg of nanorod) (Fig. 5). AgNR had been reported to have similar effects against *E*. *coil* ^8^, though we found it for four other bacteria along with *E. coli* ATCC 25922. Also, unlike previous report^8^, appreciable antibacterial effect was recorded with Ag nano spheres as well. It was suggested that the nanorod having plane (111) has higher electron density than spherical NPs. However, the sharp edge of the nanorod also contributed an important role in antibacterial action that supports our findings with nanorods^8^. In another work, it has been suggested that the vertex of triangular nanoprisms enabled the NPs to penetrate into the cell easily and also damage the outer membrane^9^. In the present work, antibacterial activities of both AgNP-sp and AgNRs were attributed to (111) plane, as the plane contains higher atomic density and also sharp vertexes of nanorod contributes the damages of outer cell wall of the tested bacteria. Moreover, the specific doses of the NPs were also found very important in enhanced antibacterial action. Figure 6 represents the time response killing activity for *K. pneumoniae* AWD5 in presence AgNP-sp and AgNR at different concentrations, including MIC values of NPs. This range of concentration was selected on the basis of initial screening where killing phenomenon was most prominent. The study revealed that the killing activity for microorganism (*K*. *pneumoniae* AWD5) was found to sustain after 240 minutes at concentrations 197 and 207 µg/ml for spherical and 320 and 720 µg/ml for rod shaped NPs as minimum cell growth was observed after 240 minutes. While maximum log reduction (6.9 to 2) was found during 60–210 min with the percentage decrease in cell population 71% at exposure of 197 µg/ml AgNP-sp. Similarly, for exposure at 184 and 207 µg/ml of AgNP-sp, log reduction value was found as 8.0–5.86 and 6.5–2.86 along with percentage decrease in cell population 26.75 and 56.90% respectively. In case AgNR, maximum log reduction (8.89–5.1) had been observed at concentration 720 µg/ml with decrease in cell population 42.63% during the same time interval. While for 560 and 320 µg/ml of AgNR, the log reduction value along with percentage decrease in cell population was observed at 8.63–6.97, 19.23% and 8.89–6.23, 29.9% respectively.

**Figure 7:**
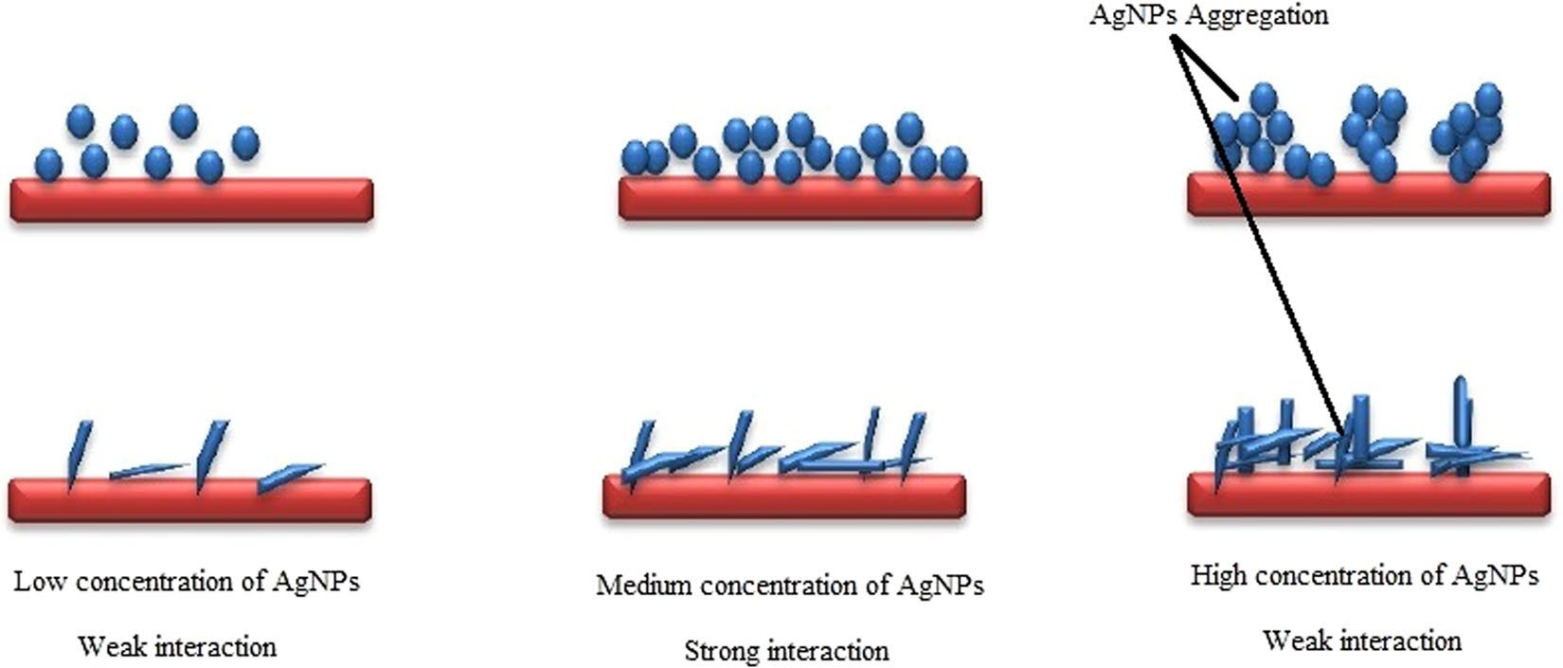
Schematic representation of the release of AgNP-sp and AgNR at lower, medium and high concentrations.

Figure 6 also depicts the percent decrease in cell population of microorganism exposed to different shaped NPs, with respect to initial cell population (0 h). Here, it was evident that the death rate of bacterium exposed to AgNP-sp (≥78%) was significantly (P < 0.05) higher as compared to AgNR (≥46%). The rate of bacterial death was higher at higher concentrations of AgNP-sp. The highest rate of cell death was recorded between 210–240 minutes at 197 μg of AgNP-sp. In AgNR, the death rate of bacterial cells was maximum between 180–210 min, for all three concentrations experimented, maximum being at 720 μg/ml. So, it was concluded that that AgNP-sp achieved faster bactericidal action within 240 minutes as compared to AgNR, that further confirms the qualitative data obtained during disc diffusion assay. In fact, the dose dependent killing kinetics of AgNPs are rarely available in literature. Earlier, dose dependent effect of AgNPs on the Gram-positive and Gram-negative bacteria has been reported on the basis of disc diffusion method^24^, where it was suggested, that at low concentrations of NPs, the interaction of particles with the cell wall of bacteria decreases, while at the high concentrations, aggregation probability of the particle increases causes less interactions with bacteria and NPs^24^. However, the findings of present work suggest that specific concentration is very important to get maximum interaction between the NPs and cell wall (Fig. 7). In this case, the antibacterial activities were studied against Gram-positive bacteria (*S. aureus* ATCC 25923*, B. subtilis* AST5-2) and Gram-negative bacteria (*P. aeruginosa* AL2-14B*, K. pneumoniae* AWD5 and *E. coli* ATCC 25922) at the different doses of AgNP-sp (230–255 µg) and AgNRs (364–399 µg). As the result obtained by disc-diffusion test, 242 µg and 249 µg was found an effective dose of AgNP-sp while 392 µg and 380 µg for AgNRs were found an effective dose against all tested bacteria respectively (Fig. 5). In another report, time assay killing kinetic test for AgNPs revealed the effectiveness in killing of tested bacteria within 180 minutes^22^. Earlier, it was reported that the minimum time necessary to achieve bacteriostatic effect ≥99% and bactericidal effect ≥99.9% expected to fall within this duration^22^.

**Figure 8:**
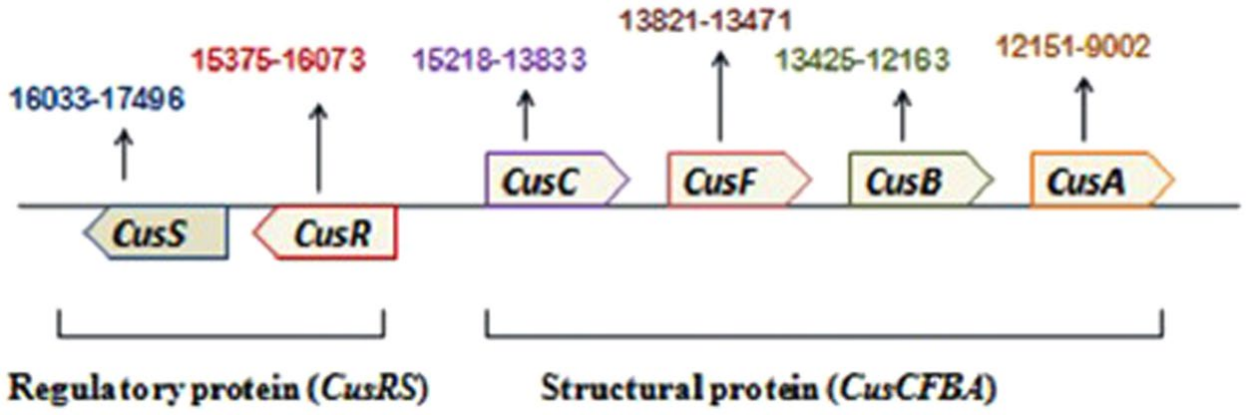
Arrangements of genes in the genome of *K. pneumoniae* AWD5 for *CusCFBA* and *CusRS* operons.

*K. pneumoniae* AWD5 strain was found to be resistant to silver ions (MIC > 500 µg/ml AgNO_3_, which is higher to MIC values of AgNP). The genome analysis and its annotations of *K. pneumoniae* AWD5 strain revealed the presence of two operons that regulates the resistance against silver and copper (Fig. 8). The *Cus* determinant of *K. pneumoniae* AWD5 comprised two operons, one structural (*CusCFBA*) and the other regulatory (*CusRS*). The *CusRS* operon encodes a histidine kinase, CusS, located at the inner cell membrane while *CusR* is a transcriptional regulatory protein present in cytoplasm. Transcription initiation of *cusCFBA* is dependent on the concentration of copper and silver^25^. This is the cation efflux system which mediates resistance to copper and silver. *CusF* and *CusA* are the periplasmic protein, *CusB* is cell membrane protein, *CusC* is the cell outer membrane protein. *CusA* and *CusB* were essential for Copper resistance and *CusC* and *CusF* were required for full resistance^26^. Interestingly, in spite of presence of these operons for Ag-resistance in the genome, the strain of *K. pneumoniae* AWD5 was sensitive to AgNPs. This clearly indicates presence of an alternative mechanisms of killing induced by NPs which may be non – specific and non-dependent to the proteins of Ag-resistance.

In fact, ability of *K. pneumoniae* AWD5 to resist Ag ions, along with the presence of operons for resistance against silver and copper ions indicated that the toxicity may not be due to silver ions and therefore, killing of bacteria was mainly due to NPs. This further reinforced the observation, and hence conclusion that the killing effect of AgNPs is mainly due to physical mutilation, because of direct interaction of NPs with bacterial cell. This was confirmed by electron microscopy data. The interaction silver of AgNP-sp and AgNRs with bacteria was studied by FE-SEM.

**Figure 9:**
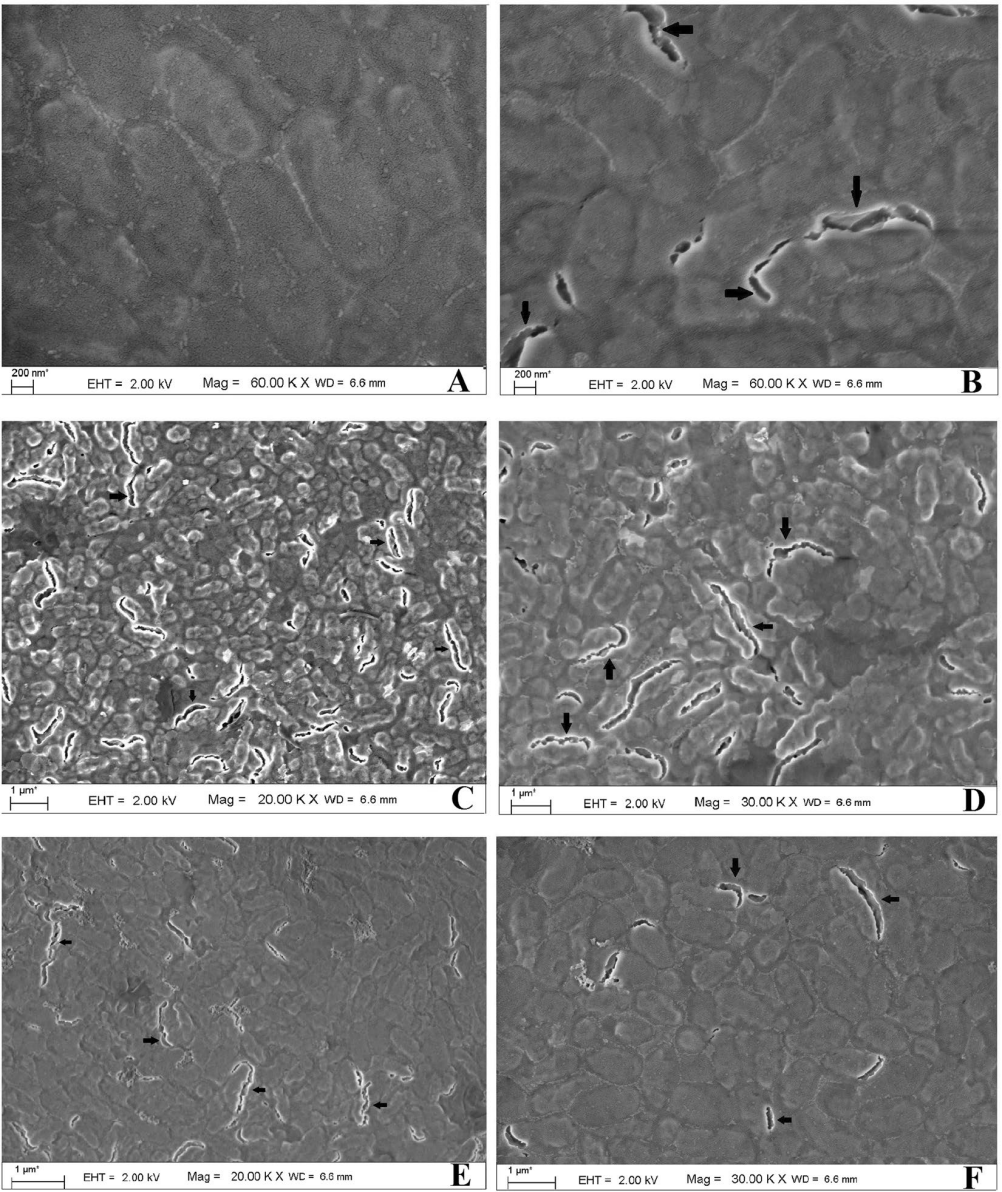
FE-SEM images of nanoparticle-untreated *K. pneumoniae* AWD5 (**A**) with intact cell surfaces, in contrast to AgNP-sp treated *K. pneumoniae* AWD5 (**B**). The cells of *K*. *pneumoniae* AWD5 were disrupted due to interaction with AgNP-sp (**C**,**D**) and AgNR (**E**,**F**). Arrows indicate disrupted regions on cell surface.

**Figure 10:**
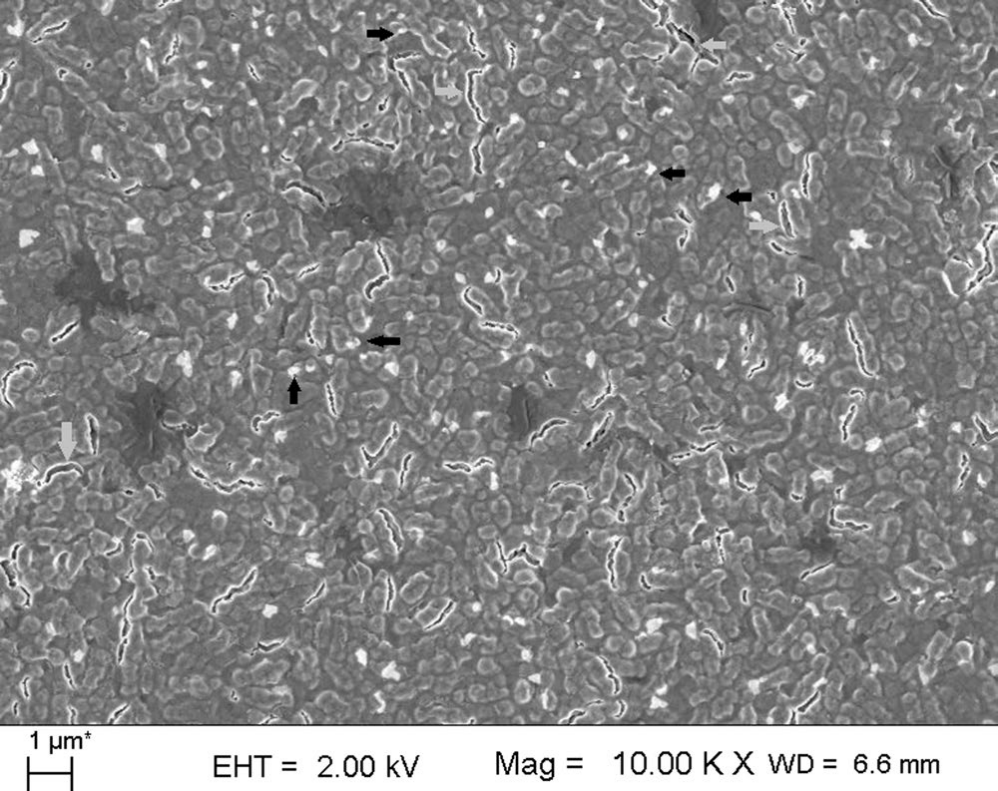
FE-SEM image showing the nanoparticles (AgNP-sp) (black arrow), overlaid with cells of *K. pneumoniae* AWD5 with damaged cell surfaces and disrupted cells (grey arrows) due to interaction with NPs.

The damaged outer wall of the bacterium (*K. pneumoniae* AWD5) is clearly visible in Fig. 9(B–F). The cell walls were observed to be disrupted and split open that might had disturbed the integrity of cell resulting in cell damage and death. Figure 9(E,F) shows the distortion of bacterial cell wall by nanorod and that can be attributed to the sharp vertex of nanorod. One high resolution representative micrograph of undamaged and damaged bacterium can be seen in Fig. 9(A,B). The rupture of outer wall confirmed the antibacterial action of NPs. The planes (111) of both shaped NPs were also assumed to be responsible for the antibacterial action. Some spherical NPs of sizes <100 nm are visible in micrograph along with few NPs attached on the bacterial surface indicated by arrow (grey color) (Figs 9C and 10). Figure 9(E,F) shows the distortion of bacterial cell wall by nanorod and that can be attributed to the sharp vertex of nanorod, as evident from TEM photomicrograph (Fig. 3C). As shown (Fig. 9A), untreated *K. pneumoniae* AWD5 appeared rod shaped with smooth and intact cell wall. After exposer to AgNPs for 3 h, the physical mutilation in bacteria was observed as evidenced by damages of cell wall which is similar to previous reports (Fig. 9B)^27,28^. This further confirmed the hypothesis on bactericidal action of AgNPs^8,27,28^.

Further, AgNP-sp treated *K. pneumoniae* AWD5 cells were observed to be more damaged, as compared to AgNR treated cells. This also substantiate the difference in respective MIC values, which was lower for AgNP-sp than AgNR. Similarly, the killing kinetic values and ZOI were relatively better for AgNP-sp. In fact, the greater distortion of bacterial cell wall by Ag nanosphere may be attributed to the granulate shape with larger surface area, as compared to one-dimension nanostructure (Ag nanorod) with low specific surface area. Recent studies confirmed that the low specific surface area of one-dimensional nanostructure like nanowires and nanorod loosely contact with bacterial cell as compared to nanocubes. Also, nanosphere that has larger effective specific contact area, achieve closer contact with bacterial cell and causes more damages^11,12^. It is relevant to mention that, the bacterial cell wall, and not cell membrane, is the first site of physical interaction with NPs. Therefore, it is more likely that the damage is induced at the cell wall of bacteria, as evident from FE-SEM data given in this study (Fig. 9). In the batch studies of disc-diffusion test, Gram-negative bacteria were found to be more affected as comparison to Gram-positive bacteria by both types of AgNPs. This may be due to the different membrane structure of the microorganism. It is well known that the Gram-negative bacteria have outer membranes of lipopolysaccharide, followed by a thin peptidoglycan layer (2–3 nm). While Gram-positive bacteria lacks the outer membrane, but have thick layer of peptidoglycan (about 30 nm), consisting of linear lipopolysaccharide chains cross-linked by short peptides which makes them rigid and less permeable. Herein, the electrostatic attraction between AgNPs and bacterial surface has been considered important for antibacterial activity. In literature, it has been suggested that the negatively charged AgNPs (citrate stabilized) interact more strongly with the positively charged residues of the integral membrane proteins on the bacteria surface and causes alteration in structural integrity or physiochemical changes in the bacteria cell wall resulting in leakage of cytoplasmic contents that ultimately cause cell death^22,29^, However, in this work, it is proposed that the physical interactions also have significant role in disruption of the bacterial cell wall as supported by FE-SEM analysis data^27^. Earlier, Li *et al*. reported damages in bacterial membrane due to metal NPs. Some of the previous reports have emphasized on role of positive charge on AgNPs in interaction with negatively charged bacterial cell wall^27^, however, the presence of capping agents (negatively charged) also need to be considered^30^.

Based on the above observations, it can be concluded that the toxicity of the NPs against various Gram-positive and Gram-negative bacteria depends on the sharp edges, vertexes and the specific doses to penetrate in to the cell wall. The exact mechanism behind bactericidal effect of AgNPs is still not known and need study further.

## Conclusions

The AgNP-sp and AgNRs were synthesized by chemical reduction method. The optical properties were studied by UV-Vis spectroscopy whereas the structure and morphological properties were characterized by FE-SEM, TEM and XRD techniques respectively. Disc diffusion method was conducted to test the antibacterial activity against Gram-positive and Gram-negative bacteria. The time-assay killing kinetic revealed that AgNP-sp and AgNR has greater kinetic effect of killing bacteria at specific doses of NPs. It was concluded that the (111) plane of both AgNP-sp and AgNRs is responsible for antibacterial effect. This was further confirmed by killing kinetics study, and supported by arrangement of Ag-resistant genes in genome of test organism.

## Methods

### Synthesis of AgNP-sp

Silver nano spheres were prepared by well-known citrate thermal reduction method at temperature 100 °C. In this synthesis process, 50 ml aqueous solution was prepared by adding 0.041 g of AgNO_3_ (Fisher scientific) (4.82 mM). Then 25 ml of prepared solution was added to 100 ml of H_2_O (deionized). The resulting 125 ml solution was then set to boil for 10–15 minutes followed by stirring on magnetic stirrer at the rate of 200 r/min. A 1% solution of C_6_H_5_Na_3_O_7_ (Fisher scientific) (0.3 g in 30 ml) was added as soon as boiling commence. The resulting solution was found light yellow in color after 15 minutes. The overall synthesis procedure was protected from light. The solution was then allowed to cool at room temperature. The AgNP-sp were further purified by centrifugation at 10000 r/min for 20 min at 27 °C and a dried powder of the nanosized silver was obtained by freeze-dying as described by Abdelwahed *et al*.^31^. To remove the excess silver ions, the silver pellet was washed three to four times with deionized water by centrifugation (10000 r/min for 20 min). The re-suspended aqueous homogenate of NPs were diluted for different trials. The original concentrations of stock solution, and dilution factors were considered for determination of concentrations of different type of AgNP-sp.

### Synthesis of AgNR

Rod shaped AgNPs were synthesized by surfactant less wet chemical method^13^. The synthesis involved two different solutions A and B. Solution A consist of 100 ml of deionized water containing 3 µl of 1 M NaOH (Fisher scientific) solution (0.8 g of NaOH in 20 ml H_2_O), 40 µl of 47.09 mM AgNO_3_ solution (0.16 g of AgNO_3_ in 20 ml H_2_O) and 5 ml of 10 mM C_6_H_5_Na_3_O_7_ solution (0.0516 g of C_6_H_5_Na_3_O_7_ in 20 ml H_2_O). Solution B was prepared by mixing of 50 ml deionized water, 3 µl of 1 M NaOH solution and 30 µl of 0.1 M AgNO_3_ solution. Solution A was heated upto boiling for 10 min followed by stirring at 500 r/min on magnetic stirrer, and solution B was added to it. The resulting solution was allowed to boil at 100 °C with rapid stirring (600 r/min). After 30 min, color of the solution was found pale yellow, suggesting the formation of AgNRs. The NPs solution was then cooled to room temperature (27 °C). The AgNRs were purified by centrifugation at 10000 r/min for 20 min at 27 °C and a dried powder of the AgNRs was obtained by freeze-dying, washed and diluted, as described above for AgNP-sp. The original concentrations of stock solution, and dilution factors were considered for determination of concentrations of different type of AgNP-sp.

### Characterization of NPs

The morphology of the synthesized NPs was studied by FE-SEM (Zeiss) and TEM (JEOL JEM 2100). The samples for FE-SEM and TEM analysis were prepared by placing a drop of homogeneous suspension on a copper grid film and allowing to dry it at room temperature. The crystalline phase and structure of the AgNPs were further analyzed by XRD spectrometer by taking small amount of solution and drying it on a quartz plates. The crystal structures of the nanoparticles were examined by Rigaku Corporation Japan/Model: Miniflex XRD with CuKα radiation (λ = 1.54056 Å). The zeta potential of AgNPs was measured by DLS with Zetasizer Nano. The optical properties of the NPs were characterized by UV-Vis (Perkin Elmer, Lamda 35) with a 1 cm quartz cell at room temperature.

### Microorganisms and Growth Conditions

Luria Bertani (LB), and nutrient medium (NM) and constituents of growth media were purchased from HI Media. *E. coli* ATCC 25922 and *S. aureus* ATCC 25923 are type strains, which were taken from culture collection of department of Microbiology, while *B. subtilis* AST5-2, *P. aeruginosa* AL2-14B^32^ and *K. pneumoniae* AWD5^33^, were isolated in an unrelated work, and identified by 16S rDNA sequence analysis. *P. aeruginosa* AL2-14b was plant endophyte while *B. subtilis* AST5-2 and *K. pneumoniae* AWD5 were isolated from soil. The bacterial strains were grown on LB or NM as per the requirements, and stored on nutrient agar (NA) slants at −20 °C. The cultures were revived on LB broth and sub-cultured after regular intervals.

### Disc diffusion test

Bacterial sensitivity to antibacterial compound is tested using a disc diffusion test, employing antibacterial compound impregnated disc. Therefore, disc diffusion assay with NPs laden disc was used in this study. The NPs laden filter paper was dried at room temperature and small disc of uniform size (5 mm diameter) containing 255, 249, 242 and 230 µg assigned as S0, S1, S2 and S3 for AgNP-sp and 399, 392, 380, 364 µg for AgNR were assigned as R0, R1, R2 and R3 respectively were punched out and stored in a desiccator at room temperature. The bacterial suspension was applied uniformly on the surface of a nutrient agar plate using sterile swab, before placing the disc on the plate (4 per plate). The bacterial density was adjusted in sterile PBS solution to get the 10^8^–10^9^ CFU/ml of cells, as adjusted by measuring absorbance at 600 nm as per Mac Farland standards. The plates were incubated at 37 °C for 24 h, after which the average diameter of the ZOI surrounding the disc was measured with a ruler with up to 1 mm resolution.

### MIC estimation

The minimum inhibitory concentration (MIC), defined as the lowest concentration of the material that inhibits the growth of microorganisms and was determined in the present report based on batch culture containing different concentrations of AgNPs in suspension. The procedure for MIC estimation was followed as per Li *et al*.^27^. Firstly, all strains were cultured and harvested by centrifugation (8000 g at 4 °C for 10 min), washed thrice in PBS (0.2 molL^−1^ at pH 7.4) and resuspended at a final concentration to maintain initial concentration 10^8^ cells/mL of PBS. The resuspended cells were inoculated into fresh medium (1 ml) supplemented with various concentration of NPs (184–195 µg/ml for AgNP-sp and 320–358 µg/ml for AgNR) and then incubated in an orbital shaker at 200 rpm at 37 °C and incubated for 24 h. Bacterial growth was measured as absorbance at 600 nm determined using a spectrophotometer (Thermo Spectronic, Helios Epsilon, USA). The experiments also include a positive control (flask containing NPs and nutrient medium without bacterial inoculums) and negative control (flask containing inoculum and nutrient media, without NPs)^34^. All the experiments had been carried out in triplicate. Both AgNP-sp and AgNR at different concentrations were tested for bactericidal effect using all the microbial culture selected for the study.

### Killing kinetics of AgNPs against *K. pneumoniae* AWD5

The dose dependent killing activity for *K. pneumoniae* AWD5 (in 20 ml PBS inoculated with 10^8^–10^9^ CFU/ml of cells) containing different concentrations of spherical (184–207 µg/ml) and rod shaped AgNPs (320–720 µg/ml) was determined. *K. pneumoniae* AWD5 was selected for study of nano-silver induced killing kinetics, because of its excellent response in antibacterial activity experiment and resistance for silver ions. Cells were grown in NB medium, at 37 °C for 24 h with continuous agitation. The cells in late log phase were harvested by centrifugation (10000 r/min, 10 min). Cells were washed thrice by centrifugation (10000 r/min, 10 min) and suspended in PBS (1.0 M, pH 8.0). The cell suspension was suitably diluted in sterilized PBS solution to get the 10^8^–10^9^ CFU/ml of cells, as adjusted by measuring absorbance at 600 nm as per Mac Farland standards, further verified by plating and enumerating CFU on nutrient agar plates. Different concentrations of AgNP-sp or AgNR were added to the PBS suspensions of bacterial cells. Culture samples were collected after 0–240 minutes of incubation and spread onto NA agar plates. Colony-forming units were counted after incubation at 37 °C for 24 h, and percent-decrease of cell population was determined. The viable cell count of test organism was estimated in parallel, experimented without any AgNPs, which served as control.

### Analysis of *K. pneumoniae* genome for metal resistance elements

The complete genomic DNA from *K*. *pneumoniae* AWD5 was sequenced using next generation sequencing system. The libraries were sequenced using the HiSeq. 2500 platform (Illumina, San Diego, USA). The sequences of the genome were assembled with SOAPdenovo2. The genome was assembled into137contigs using CLC Genomics Workbench v9.0. The gene identification and annotation were provided by NCBI, conducted by using NCBI Prokaryotic Genome Annotation Pipeline, with Best-placed reference protein set; Gene MarkS+ annotation method. The draft genome was also annotated using BASys bacterial annotation system. This whole-genome shotgun of *K. pneumoniae* AWD5 has been deposited at Gen Bank under Bio project ID PRJNA326255 with accession no. MOXK00000000.

### FE-SEM analysis for interaction of Bacteria with AgNPs

50 ml of Log phase culture of *K*. *pneumoniae* AWD5 was treated with already prepared NPs, and incubated for 3 h. The concentration of NPs was adjusted to respective MIC values, and cells were exposed till 3 h as per the results of time-assay killing kinetics data. Pellets were collected and washed two - three times by centrifugation (10000 r/min for 10 min) and resuspended in PBS to achieve 10^8^ CFU/ml as described above. The cells were fixed for 3 h in 2.5% glutaraldehyde at room temperature (~27 °C) and coated with gold followed by deposition on copper grid for observation. The interactions of the AgNPs with bacteria (*K. pneumoniae* AWD5) cells were examined using FE-SEM. Cells not exposed to NPs were processed in parallel to serve as control.

### Statistical analysis

The experimental data for MIC and time assay killing kinetic assay were statistically analyzed. Standard error of means was calculated following the standard procedure and compared for significance by independent t-test at P < 0.05 using SPSS 16 software. Population densities of the isolates were further estimated using log.
